# Metallic versus Non-Metallic Cerclage Cables System in Periprosthetic Hip Fracture Treatment: Single-Institution Experience at a Minimum 1-Year Follow-Up

**DOI:** 10.3390/jcm11061608

**Published:** 2022-03-14

**Authors:** Attilio Speranza, Carlo Massafra, Stefano Pecchia, Riccardo Di Niccolo, Raffaele Iorio, Andrea Ferretti

**Affiliations:** Orthopaedic and Traumatology Unit, Sant’ Andrea Hospital, “Sapienza” University of Rome, 00189 Rome, Italy; atsperanza@gmail.com (A.S.); ste.pecchia@gmail.com (S.P.); riccardo.diniccolo@gmail.com (R.D.N.); raffaele.iorio@uniroma1.it (R.I.); aferretti51@virgilio.it (A.F.)

**Keywords:** periprosthetic fracture, periprosthetic treatment, hip arthroplasty, cerclage cables, fracture healing

## Abstract

Metallic cerclage cables are reliable and cost-effective internal fixation devices, which are largely used in surgical practice for the treatment of periprosthetic fractures. Nevertheless, complications connected with their use have been described in the literature, including the following: third-body generation, failure and consequent migration, fraying, allergies, and injury to the surgical team. The development of new materials offers alternatives to traditional metallic cables. This study compares the outcomes between two groups of patients affected by periprosthetic hip fractures, treated with titanium cables or with ultra-high-molecular-weight polyethylene (UHMWPe) iso-elastic cables. Our retrospective study aims to compare the clinical and radiological outcomes of titanium cables and UHMWPe iso-elastic cables, isolated or associated with dedicated plates, for the surgical treatment of periprosthetic fractures with stable implants. Two groups of 30 (group A—metallic cables) and 24 (group B—UHMWPe iso-elastic cables) patients have been surgically treated in our institution for hip periprosthetic fractures, between September 2017 and June 2020. The mean age of the patients was 81 years in group A and 80 years in group B. In our study, we included fractures classified as B1 or C, according to the Vancouver postoperative fractures classification; the patients were evaluated retrospectively at 1 year postoperatively, regarding the following: surgery time, blood loss, partial weight-bearing time, radiographical healing time, Harris hip score, and postoperative complications. Comparable outcomes were observed in patients from both groups. Group A showed a higher complication rate compared to group B, at 1 year postoperatively. Non-metallic nylon fiber and ultra-high-molecular-weight polyethylene (UHMWPe) cerclage cables could represent a reliable fixation device, ensuring comparable healing and complication rates with traditional titanium cerclage cables.

## 1. Introduction

Total hip arthroplasty (THA), as one of the most effective and safe surgical procedures, is becoming more commonly performed. In 2017, the United Nations reported that 25% of the European population is aged 60 years or older, and the proportion will rise to 35% by 2050 [1]; moreover, osteoporosis, which shows a higher prevalence in older patients, has been shown to be a major risk for hip fractures [2]. An ageing population, with high life quality and subsequently higher functional demand compared to the past, is leading to a rise in the primary THA rate. Recent studies showed a 50% increase in the primary total hip arthroplasty rate from 1990 to 2002, and a 60% increase in revision hip arthroplasty in the US [3], while the projection of time trend in the UK revealed a 40% increased rate by the year 2030 [4]. While, generally, the complication rates for THA are low, periprosthetic hip fractures are a severe complication after THA, and often occur several years after the primary procedure. In recent years, in parallel with the trends previously discussed, cases are increasingly common in the general population. Surgical treatment is demanding, and often leads to high complication rates and poor prognosis, concerning outcomes and mortality [5,6]. The cerclage technique is a simple fixation method, involving a cable or a wire looped and tightened around a bony structure to provide stability; it is crucial to identify the preferred surgical technique and internal fixation device material to obtain the optimal results. The correct indications must consider the fracture type, implant stability, bone stock quality, preexisting infections, and patients’ general clinical conditions. The Vancouver classification is widely used to assess treatment strategies, depending on the fracture type; the features considered are fracture location, implant stability, and bone stock quality. The different surgical techniques, hardware, and indications of implant revision and bone grafting are described [7]. B1 and C fractures with a stable primary implant can be treated with cerclage wires and cables alone, but locking plates are commonly used in association. Recent evidence suggests a role for open reduction internal fixation (ORIF), even in reducible B2 fractures with an intact cement mantle [8], and in B3 fractures [9]. The surgeons’ choice regarding cerclage hardware is mainly between the following two categories: metallic or non-metallic. Metallic wires or cables commonly consist of titanium or stainless steel hardware, differently configurated as single wire, cable, or braided [10]. Metallic materials have classically been widely used in clinical practice, with good results, and are considered a reliable and versatile internal fixation device. The technique involves the preparation of the bony structure and the use of a wire passer to minimize soft tissue damage. The cable is then tensioned and fixed with specific tools. Serious complications linked to metallic wires have been described in the literature, including the following: third-body generation, failure and consequent migration, fraying, allergy, non-union, and injury to the surgical team [11]. Non-metallic cerclage can be made of absorbable or non-absorbable suture material, used as a simple suture or with more complex configurations. Several authors have been investigating the reliability of non-metallic materials for the treatment of different anatomical districts, showing interesting results [12,13,14,15]. Periprosthetic fracture patients often suffer important morbidities and mortality, and stability and early mobilization are critical to lower the complication rates as much as possible. We present a comparison of clinical outcomes between two groups of patients suffering from periprosthetic hip fractures with stable implants (Vancouver B1 and C). The first group was treated with traditional metallic cables, whereas the second group was treated with UHMWPe cables. The purpose of this study is to compare outcomes and reliability between the two alternative cerclage devices.

## 2. Materials and Methods

**Figure 1:**
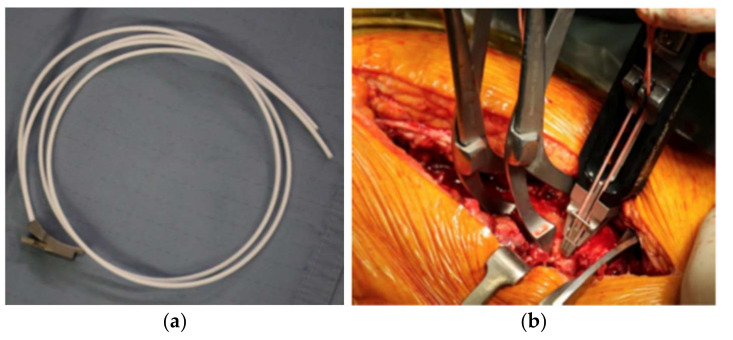
(**a**) UHMWPe cerclage cables (SuperCable^TM^; Kinamed Inc., Camarillo, CA, USA); (**b**) tensioning instrument allows for tightening and locking of cables.

**Figure 2:**
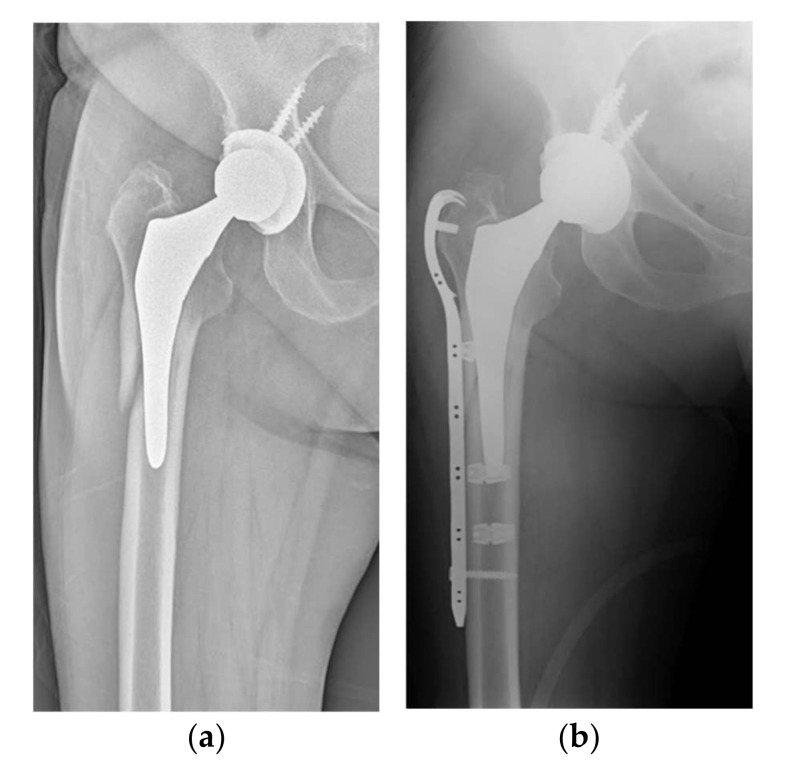
(**a**) Preoperative X-ray of type B1 fracture in 76-year-old patient; (**b**) postoperative 3-month follow-up after placement of a plate with UHMWPe cables.

Sixty-two patients have been surgically treated in our institution for hip periprosthetic fractures between September 2017 and June 2020. All demographic data are shown in Table 1 (demographic data, including age, sex, BMI, and fracture type, were recorded). All patients included in our study had reported fractures classified as B1 or C, according to the Vancouver postoperative fractures classification; fractures involving the trochanteric alone (A), loose implants (B2), and poor bone stock (B3) have been excluded (Table 2). A minimum of 1 year radiographic and clinical follow-up was required to be included in the study. All fractures were evaluated by means of a preoperative standard anteroposterior (AP) radiograph of the pelvis and a lateral view of the hip. In all cases, the procedure was performed by a single experienced surgeon. Patients were divided into two groups of 32 (group A) and 30 (group B), according to the type of cerclage used, either metallic cerclages (group A) or UHMWPe cerclage cables (group B) (SuperCable^TM^; Kinamed Inc., Camarillo, CA, USA). The cables consist of a nylon core surrounded by UHMWPe braided fibers, fixed with a metallic clamp. Cables were passed with a semicircular passer and then stabilized using the provided tensioning device (Figure 1). Metallic cerclages were used with a dedicated plate system (Zimmer Inc., Warsaw, IN, USA). A dedicated plate was also used for UHMWPe cables (SuperCable Grip and Plate System^TM^; Kinamed Inc., Camarillo, CA, USA) (Figure 2). A standard lateral approach to the hip was performed in all cases, with patients lying in lateral decubitus. The stability of femoral and acetabular components, and the integrity of the articular surface, were tested during surgery; patients with unstable components underwent THA revision and were excluded from this study. The incision was expanded as needed to obtain satisfactory exposure of the fracture site with minimal periosteal stripping. Patients were evaluated for the following surgery-related factors: operation time, blood loss on third day, and surgeon injury. At 1 year postoperatively, the following factors were evaluated: fracture healing time, Harris hip score at 1-year follow-up, and postoperative complications. Radiographic non-union was defined as the persistence of the fracture line through cortical bone on two different views. Hardware failure was defined as implant deformation or breakage evidence on follow-up plain X-rays. Fracture healing was based upon bridging bone on standard-view X-rays, absence of migration greater than 3 mm, absence of implant failure, and absence of pain with weightbearing. Two observers’ consensus was needed to define radiographical features. Postoperatively, all patients were prescribed partial weight bearing, and the rehabilitation protocol was carried out at specialized institutions. The data were analyzed using IBM SPSS Statistics for Windows, version 23.0 (IBM Corp., Armonk, NY, USA). All the data were first analyzed for normality of distribution using the Kolmogorov-Smirnov test. The variables were expressed as means (standard deviation). Student’s t-test was performed and *p* < 0.05 was considered to be significant.

## 3. Results

Of the 62 patients included in the study, 4 were lost to follow-up. Five patients were excluded because they were treated using cerclages alone, without plates—two in group A and three in group B. The analysis was then conducted on 28 patients in group A and 25 in group B. The mean follow-up was 15.7 months (range 12–18 months). The mean age of the 62 patients was 76.8 years (75.1 years in group A vs. 77.3 years in group B; *p* > 0.05). The mean operative time was 87.3 ± 16.2 min in group A and 83.6 ± 17.3 min in group B (*p* > 0.05), with a mean hemoglobin loss on the third day of 2.6 ± 1.4 g/dL in group A versus 2.9 ± 1.3 g/dL in group B (*p* > 0.05). The fractures showed full radiographical and clinical healing at a mean of 12.6 ± 4.3 weeks postoperative in group A and 12.8 ± 2.7 weeks postoperative in group B (*p* > 0.05). Among the patients treated with metallic cables, we reported two cases of cable rupture, two cases of osteolysis, and one case of non-union. In the UHMWPe cable group, we reported one case of THA posterior dislocation, treated with closed reduction and rehabilitation, one case of superficial wound infection, treated with antibiotics and surgical debridement, and one case of malunion. During surgery in the metallic group, there were three surgeon injuries, while no events occurred in the UHMWPe group. The mean Harris hip score at the 12-month follow-up was 72.5 ± 10.1 in group A and 72.9 ± 8.4 in group B. No statistically significant differences were found between the groups regarding the data considered.

## 4. Discussion

This study investigates clinical and radiological outcomes in patients treated for periprosthetic fractures with metallic and UHMWPe cerclage cables; the results show that UHMWPe cables yield comparable outcomes when compared to traditional metallic devices. The goal of surgical treatment of periprosthetic fractures is to ensure early bony stability, thereby healing the fracture and restoring limb function [16]. Although there is a variety of possible treatments, type B fractures still present a challenge, because these fractures involve the bone in the vicinity of the femoral stem. Fractures with a well-fixed stem (Vancouver type B1) are commonly treated by open reduction and internal fixation [17]. The variety of methods, implants, and their combinations means that no ‘‘gold standard’’ exists. Currently, there is evidence to support the use of the following three common constructs: plate-cable systems, locking plates, and compression plates; no single method demonstrates superiority in all cases [18]. Fixation that is too rigid can be a problem, as some degree of movement is necessary for callus formation, and increasing stiffness of a construct can lead to mechanical fatigue [19]. Biomechanical studies suggest that plate-cable constructs with proximal unicortical screws alone, or unicortical screws with cables, provide added stability in compression, lateral bending, and torsion, compared to proximal cables alone [20]. Locking plates are particularly suitable for fixation in patients with poor bone quality, such as patients with periprosthetic fractures. The use of cerclages and cables has been controversial. Cerclages alone in the treatment of these fractures does not provide sufficient strength and does not allow for early mobilization in these complex patients, but can considerably contribute to greater stability and longer fixation when combined with the use of locking plates [16]. In recent times, device development, boosted by the increasing incidence of periprosthetic fractures, and by an ageing population, has brought new materials and novel hardware to the surgeon’s options. Aside from the biomechanical data provided by the literature, clinical evidence is needed to determine the correct indications and suitability of the new materials for optimal results, in terms of functional outcomes. By analyzing biomechanically metallic cables and UHMWPe cables, one could intuitively state that metallic cables provide better strength and resistance. There is a plethora of definitions of strength. However, studies have shown that when considering the load to failure, that is, the load at which a loss of strength and elasticity occurs and rupture happens, as the strength parameter, non-metallic compositions provide higher load resistance in shoulder prosthetic implant stabilization or tuberosity refixation in shoulder arthroplasty [12,14]; moreover, in proximal femur cerclage, 25% less strain over time has been observed for non-metallic cables [13]. However, using Ethibond, a different non-metallic cerclage configuration for proximal humerus fractures, Knierzinger reported a lower load to failure compared to metallic cables [21]. Camarda systematically evaluated the literature regarding patella fracture osteosynthesis with non-metallic cables, reporting a 90% success rate [22]. Traditionally, various types of metallic cables have been used in hip reconstructive surgery [23]. However, their use poses several limitations and potential complications, including fraying, third-body generation, accelerated wear of the bearing surface, and injury to the surgical team [24,25]. Some studies have shown that the use of non-metallic cables may be a viable alternative in this type of surgery. The iso-elastic cable maintained compression during the time period, associated with primary bone healing, and showed a substantially superior fatigue endurance limit compared to metal cerclages [26]. In a study of 29 THA revision cases, Ting showed a nonunion rate of 7% when using the same type of implant evaluated in this study. Comparing this with other studies [11,27,28,29], it is possible to note that there is a lower rate of cerclage failure and a comparable nonunion rate. To the best of our knowledge, this represents the first study comparing a group of patients operated on for periprosthetic hip fractures with metallic cables and a group operated on with non-metallic cables (UHMWPe cables). For clinical results and fracture healing capacity, there was no statistically significant difference between the two types of cerclages. In the group of patients treated with metallic cerclages, a higher number of related complications was found; in two cases, cerclage rupture occurred, and in three cases, osteolysis was radiologically detected at the position of the cerclage. These results are in agreement with studies in the literature, which have shown that a higher percentage of metallic cerclages fail, and are subsequently removed, with a percentage ranging from 25 to 75%, depending on the anatomical site [30,31,32]. This study has some limitations. Only two subgroups of periprosthetic fractures were considered, type B1 and C, according to the Vancouver classification, where a revision of the femoral component of THA was not performed. The cohort of patients studied was small and the follow-up was relatively short, although it was sufficient to highlight some complications related to the type of cerclage used, but subsequent analysis may be necessary at a longer follow-up. Further studies with a larger group of patients and different types of fractures could be useful to highlight further useful information on the use of non-metallic cerclages.

## 5. Conclusions

Metallic and non-metallic cerclages lead to similar clinical results when used in periprosthetic hip fractures. No statistically significant difference was noted between the two groups. More complications were noted when metallic cerclages were used. The use of non-metallic cerclages in UHMWPe appeared safe and reliable for this type of fracture, representing a viable alternative for surgeon.

## Figures and Tables

**Table 1:** Demographic data.

	Group A (*n* = 32)	Group B (*n* = 30)
Age (years)	75.1 ± 8.1	77.3 ± 4.1
Sex (M; F)	13; 19	9; 21
BMI (body mass index)	25.3 ± 4.2	26.1 ± 3.7
Fracture		
B1	22	21
C	10	9
Follow-up (months)	16.1 ± 3.2	14.8 ± 2.9

**Table 2:** Vancouver classification.

Type	Subtype	Fracture Description
A		Fracture in trochanteric region
	AG	Fractures of the greater trochanter
	AL	Fractures of the lesser trochanter
B		Fracture around stem or just below it
	B1	Well-fixed stem
	B2	Loose stem with good proximal bone stock
	B3	Loose stem with poor-quality bone stock
C		Fracture occurring well below the tip of the stem
